# Social media sharing of low-quality news sources by political elites

**DOI:** 10.1093/pnasnexus/pgac186

**Published:** 2022-09-22

**Authors:** Jana Lasser, Segun Taofeek Aroyehun, Almog Simchon, Fabio Carrella, David Garcia, Stephan Lewandowsky

**Affiliations:** a Institute for Interactive Systems and Data Science, Graz University of Technology, Inffeldgasse 16C, 8010 Graz, Austria; b Complexity Science Hub Vienna, Josefstädterstraße 39, 1080 Vienna, Austria; c School of Psychological Science, University of Bristol, Bristol BS8 1TH, UK; d School of Psychological Science, University of Western Australia, 35 Stirling Hwy, Crawley, WA 6009, Australia

**Keywords:** misinformation, elites, political discourse

## Abstract

Increased sharing of untrustworthy information on social media platforms is one of the main challenges of our modern information society. Because information disseminated by political elites is known to shape citizen and media discourse, it is particularly important to examine the quality of information shared by politicians. Here, we show that from 2016 onward, members of the Republican Party in the US Congress have been increasingly sharing links to untrustworthy sources. The proportion of untrustworthy information posted by Republicans versus Democrats is diverging at an accelerating rate, and this divergence has worsened since President Biden was elected. This divergence between parties seems to be unique to the United States as it cannot be observed in other western democracies such as Germany and the United Kingdom, where left–right disparities are smaller and have remained largely constant.

## Introduction

Elite cues are important drivers of public opinions and discourse (1). For example, public concern about climate change is largely influenced by opinions and information shared by elites (2). The public’s increased polarization over climate change reflects the retreat of the Republican leadership from the scientific evidence (3). The power of elites to set the agenda of public conversations extends to mainstream media. For example, Donald Trump has been shown to successfully divert media attention away from topics that were potentially harmful to him (4). Notwithstanding the importance of elite discourse, research attention has only recently shifted to investigating the information sharing practices of a broader range of members of the political elite (5). Here, we contribute to these analyses by investigating the quality of information shared on social media by members of the US Congress. We find that the trustworthiness of information shared by members of the Republican Party is declining and that this decline has accelerated after the election of Joe Biden as president. We contrast these findings with information sharing practices of political elites in two other western democracies, Germany and the United Kingdom. We choose these countries because they are among the largest western economies that share a core bicameral political system in which the lower house is elected by the people. Nevertheless, they differ in interesting ways that may lead to different outcomes. For example, both the United States and the United Kingdom function mostly as two-party democracies, whereas Germany is a multi-party state. The United States was designed to break away from monarchy while the United Kingdom chose a more gradual approach. In addition, Germany has multiple public broadcasters ,while the United Kingdom has only one, and in the United States ,public broadcasting does not take a lead role in the media landscape. Although parties on the political right tend to share somewhat less trustworthy information in all three countries, the rapid decline of information quality shared by Republicans during the last 6 years is unique to the United States.

## Results

To assess the trustworthiness of information shared by politicians with the general public, we retrieve three corpora of tweets: by former and active members of the US Congress, the German parliament and the British parliament. For each corpus, we retrieve all tweets posted between 2016 January 1 and 2022 March 16. We do not include retweets. We extract all URLs included in the tweets. We follow an approach employed by similar research in this domain (6,7) and use a trustworthiness assessment by professional fact checkers of the domain a link points to. To this end, we use the NewsGuard database (8). As of 2022 March, NewsGuard indexes 6860 English and 145 German language domains. Each domain is scored on a scale of 0 (very poor) to 100 (exceptional quality journalism) points. Domains with less than 60 points are considered “not trustworthy” (8). The majority of indexed domains (62.8% for English and 74.5% for German) are considered trustworthy. After excluding links to social media websites and search engines, the database covers 46.5% of links posted by members of the US Congress and 58.8% and 39.2% of links posted by members of the German and British parliament, respectively. Coverage generally increases slightly over time and is similar between parties (see Supplementary Material Fig. S2 for details).

**Fig. 1:**
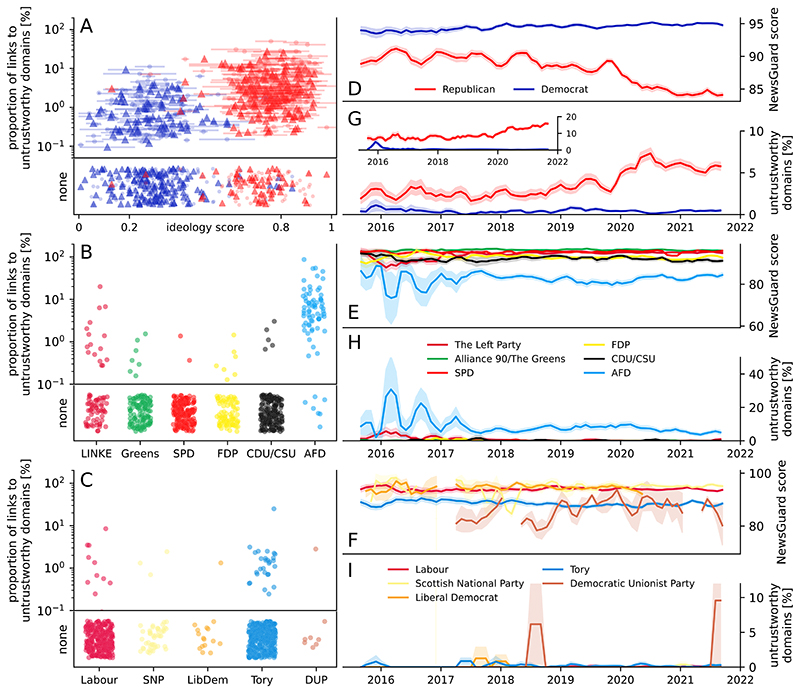
Proportion of links to untrustworthy domains posted by Twitter accounts associated with Democratic and Republican former (dots) and active (triangles) members of the US Congress (A), members of the German (B), and British parliament (C). Average NewsGuard score of links posted by members of the US Congress (D), members of the German parliament (E), and members of the British parliament (F) between 2016 and 2022. Proportion of links to untrustworthy domains posted by members of the US Congress (G), members of the German parliament (H), and members of the British parliament (I) between 2016 and 2022. The inset in G shows a reproduction of the result using an independently compiled list of untrustworthy domains (see extended methods in the online Supplementary Material). Scores and proportions of untrustworthy domains were averaged over monthly intervals with a rolling average of three months, and are broken down by party, color-coded by commonly used party colors. The 95% confidence intervals were computed with bootstrap sampling over 1,000 iterations.

We report the overall proportion of links that point to domains that are considered untrustworthy, as well as the NewsGuard score. Figure 1A to C shows the proportion of links to untrustworthy domains for the three countries. For the United States, we report values over ideology scores provided by GovTrack (9), for Germany and the United Kingdom, we report values broken down by parties. Republicans share more untrustworthy information than Democrats (note the logarithmic scale). For Germany, parties on the extreme left and extreme right share more untrustworthy information than parties in the center. Overall, Republicans share 9.1 times more links to websites considered unstrustworthy than Democrats (Republicans 3.86%, Democrats 0.43%, difference 3.44%, 1.65 SD). For Germany, members of the CDU/CSU post 6.3 times more links to such websites than members of the SPD (CDU/CSU 0.18%, SPD 0.03%, difference 0.15%, 0.04 SD). For the United Kingdom, members of the Tories post 4.7 times more links to untrustworthy domains than members of the Labour Party (Tory 0.25%, Labour 0.05%, difference 0.19%, 0.08 SD). For both Germany and the United Kingdom, the conservative parties post more links to untrustworthy domains than their counterparts on the left, but overall they post about half as many such links as the Democrats in the United States. We also note that numbers for Germany and the United Kingdom are based on very low overall counts of links to untrustworthy domains.

In Fig. 1D to F, we show the temporal trend of the NewsGuard score,averaged overall links posted in a givenmonth broken down by party. Links posted by Republicans show a notable decrease in trustworthiness, from on average 89.9 ± 0.1 (mean ± SD) points in the years 2016 to 2018 to 85.2 ± 1.8 points in the years 2020 to 2022; the score of links posted by Democrats stays remarkably stable (94.2 ± 0.4 in 2016 to 2018 and 94.8 ± 0.1 in 2020 to 2022 (interaction effect: beta =−4.01, 95% CI [−4.66, −3.37], *t*(204532) =−12.13, *P* < 0.001, see Supplementary Material Table S6 for details). This development is not reflected in the trustworthiness scores of links posted by conservative or far-right parties in other countries. In Germany, the scores of links posted by the AfD and members of the CDU/CSU stays stable with an average score of 83.9 ± 1.1 and 92.2 ± 0.8 in 2016 to 2018 and a score of 83.0 ± 1.3 and 91.6 ± 0.8 in 2020 to 2022, respectively. Similarly, the scores of the Democratic Unionist Party and Conservatives in the British parliament stay stable or slightly improve, with 83.4 ± 1.0 and 89.1 ± 0.6 in 2016 to 2018 and 85.6 ± 5.4 and 88.2 ± 0.3 in 2020 to 2022, respectively. These overall trends are also reflected in the proportion of links to domains considered untrustworthy (score <60), shown in Fig. 1(G to I). The proportion of links to these domains posted by Republicans doubles, from 2.4% ± 0.2% in 2016 to 2018 to 5.5% ± 0.7% in 2020 to 2022. The proportion of untrustworthy links posted by Democrats shows no change, from 0.4% ± 0.3% in 2016 to 2018 to 0.4% ± 0.1% in 2020 to 2022. For the German AfD, the proportion decreases from 8.3% ± 2.2% to 6.4% ± 1.9%. The proportion of links to untrustworthy domains of all other parties in Germany and the United Kingdom is similar to the proportion posted by Democrats and does not change over time.

As a robustness check, we reproduced our main result (viz. the increase in the proportion of links to untrustworthy domains by Republicans) using a second database of domain trustworthiness, compiled independently of NewsGuard (see the "Materials and Methods" section for details). The observed temporal trend of the proportion of links to untrustworthy domains is displayed in the inset of Fig. 1(G), and shows a similar trend in the proportion of untrustworthy domains posted by Republicans (from 5.9% ± 0.3% in 2016 to 2018 to 11.3% ± 2.9% in 2020 to 2022), while the proportion of links to untrustworthy domains posted by Democrats slightly decreases (0.8% ± 0.8% and 0.3% ± 0.1%).

## Discussion

Several recent analyses have shown that American conservatives are more likely to encounter and share untrustworthy information than their counterparts on the political left (6,10,11). Although the reasons for this apparent asymmetry are still debated, one possible explanation appeals to partisan motivations. Evidence suggests that derogatory content toward the political outgroup increases sharing intentions among partisans (12). According to a recent study, greater negativity toward Democrats is mostly found in lower-quality outlets, which may explain conservatives’ over-representation in sharing untrustworthy information (13). This might also explain the difference between the United States and the other countries, if right-wing news outlets outside the United States are not more negative toward the political left or if links are not the dominant mode of misinformation sharing in these countries. Another possibility is that right-wing actors leverage controversial outlets in order to get more traction on social media, as has been evident in the United States, United Kingdom, and Germany (14).

Here, we contribute to a potential explanation by showing that Republican members of Congress have become increasingly likely to share untrustworthy information on Twitter. This pattern can contribute to the observed asymmetry among the public in at least two ways: first, by directly providing misinformation to Republican partisans and, second, by legitimizing the sharing of untrustworthy information more generally. Notably, this pattern is less pronounced in two major western democracies: although politicians from mainstream conservative parties in both Germany and the United Kingdom tend to share information that is of slightly lower quality than information shared by their counter parts on the left, the gap is not large and there is no evidence of it widening. We are thus experiencing a uniquely American dilemma.

## Materials and Methods

### Twitter corpus

A corpus of tweets from former and present members of the US Congress, the German parliament, and the British parliament was collected by scraping all tweets from accounts associated with the respective politicians between 2016 January 1 and 2022 March 16. This resulted in a total of 1023 unique Twitter handles for the United States, covering 61.7% of politicians that served in the 114th, 115th, 116th, or 117th Congress. For Germany and the United Kingdom, a total of 822 (65.7% of parliamentarians) and 726 unique Twitter handles (78.6% of parliamentarians) are included, respectively (see Supplementary Material Fig. S1 and Tables S1 to S3 for details). To build the text corpus, all tweets posted by the collected Twitter accounts in the specified time frame were obtained via the Twitter API. We chose 2016 January 1, as the earliest date because before this date very few tweets by parliamentarians in Germany and the United Kingdom were available. Excluding retweets and tweets not in the country’s respective national language, the corpus contains a total of 1,694,403, 754,233, and 960,114 tweets for the United States, Germany, and United Kingdom, respectively. To determine the trustworthiness of information posted by the politicians, we extracted all URLs linking external sites contained in the tweets (shortened links such as bit.ly were expanded to determine the actual domain).

### Information trustworthiness

Following the methods of prominent research concerned with the trustworthiness of information (6,7), we use source trustworthiness as an estimator for the trustworthiness of an individual piece of shared information. Specifically, we classified the trust-worthiness of links tweeted by politicians based on the trust-worthiness of the domain rather than specific items of content. We used nutrition scores provided by NewsGuard (8), a company that offers professional fact checking as a service and curates a large data base of domains (see Supplementary Material for details). The trustworthiness of a domain is assessed on a scale of 0 to 100 points, where domains with a score of 60 or higher are labelled as “generally adhering to basic standards of credibility and transparency” (8). Similar to (15), which also uses the News-Guard data base to establish domain trustworthiness, we use this value as a threshold below, which we classify a domain as “not trustworthy”.

To validate our use of NewsGuard, we investigated whether any major news outlets are missed by the data base and whether missed news outlets have a political bias (see Supplementary Material). To further validate our use of NewsGuard, we compiled an independent data base of domain trustworthiness from a range of academic and journalistic fact-checking sites (see Supplementary Material Tables S4 to S5 for details).

## Supplementary Material

Supplementary material

## Data Availability

Full reproduction materials, including data (tweet IDs, party labels, and independently compiled list of domain labels) and analysis code, but excluding the NewsGuard data base, which is proprietary are accessible at https://doi.org/10.17605/OSF.IO/MQHGP. To acquire data from NewsGuard, contact support@newsguardtech.com.

## Significance Statement

It is widely acknowledged that what politicians share on social media helps shape public perceptions and views. Although there has been an explosion of research on the effects of misinformation and how it might best be corrected, the behavior of politicians in disseminating false or untrustworthy information has thus far largely escaped research attention. This study analyses 3.4 million tweets from U.S. American, British and German politicians made between 2016 to 2022. Conservative politicians share information of lower quality than their liberal counterparts and Republican members of the U.S. Congress are increasingly circulating news from dubious sources over the last four years. These results highlight the contribution of political elites in polluting our online environments.
