# Correction to: Social media sharing of low-quality news sources by political elites

**DOI:** 10.1093/pnasnexus/pgad158

**Published:** 2023-05-18

This is a correction to: Jana Lasser, Segun Taofeek Aroyehun, Almog Simchon, Fabio Carrella, David Garcia, Stephan Lewandowsky, Social media sharing of low-quality news sources by political elites, *PNAS Nexus*, Volume 1, Issue 4, September 2022, pgac186, https://doi.org/10.1093/pnasnexus/pgac186

In the originally published version of this manuscript, the following sentence in the Materials and Methods section, under subheading ‘Twitter corpus’, required a correction: ‘A corpus of tweets from former and present members of the US Congress, the German parliament, and the British parliament was collected by scraping all tweets from accounts associated with the respective politicians between 2016 January 1 and 2022 March 16’.

The sentence now reads as follows: ‘A corpus of tweets from former and present members of the US Congress, the German parliament, and the British parliament was collected by scraping the most recent 3,200 tweets from accounts associated with the respective politicians between 2016 January 1 and 2022 March 16 (an additional analysis for all tweets is reported in the supplement which does not yield appreciably different results)’.

The Supplementary data file accompanying the manuscript has also been updated.

