# Supplementary material for: Social media sharing of low-quality news sources by political elites
Source: PNAS Nexus. Author manuscript; Available in PMC 2022 Nov 11. (PMC7613815; doi:10.1093/pnasnexus/pgac186)
Supplement: Supplementary file 1 [file EMS156631-supplement-Supplementary_material.zip › PNASNEXUS-PNASNEXUS-2022-00579-T-s01.pdf]

Supplementary Information for

Social media discourse of political elites: An asymmetric American exceptionalism

Jana Lasser, Segun Aroyehun, Almog Simchon, Fabio Carrella, David Garcia, and Stephan Lewandowsky

almog.simchon@bristol.ac.uk

This PDF file includes:

Supplementary text
Figs. S1 to S2
Tables S1 to S6
SI References

Supporting Information Text

Extended methods.

Politician Twitter accounts. A corpus of tweets from former and present members of the U.S. Congress, the German parliament and the British parliament was collected by scraping all tweets from accounts associated with the respective politicians between January 1, 2016 and March 16, 2022. The number of tweets over time broken down by different parties for the three countries is shown in Fig. S1.

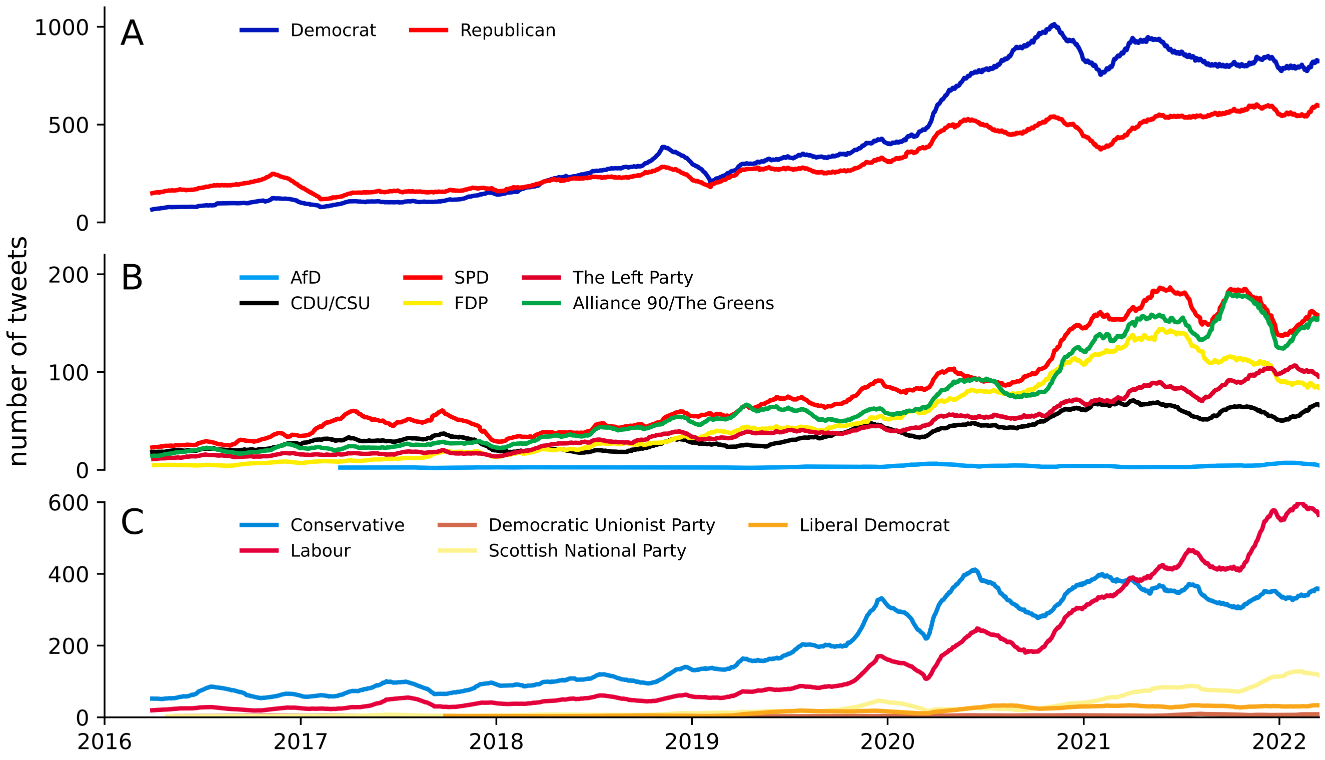

Fig. S1. Number of tweets per day posted by politicians for **A** U.S. Congress Members, **B** members of the German parliament and **C** members of the British parliament.

For the U.S., lists of Twitter handles of the 114th to 117th Congress were collected from a number of sources[*][†][‡]. This resulted in a total of 1023 unique Twitter handles, which includes congressional staff and congress member campaigns. These accounts cover a total of 504 unique Congress Members out of 817 Congress Members serving in the 114th to 117th Congress. Coverage by party and election period is shown in Tab. S1.

party	114th Congress (2015-17)	115th Congress (2017-2019)	116th Congress (2019-2021)	117th Congress (since 2021)
total	63.1%	70.4%	83.9%	82.1%
Democrats	72.8%%	78.6%	84.1%	87.5%
Republicans	54.9%	62.9%	82.7%	76.4%

Table S1. Share of U.S. Congress Members that have a Twitter account and are included in the data set split by election period and party.

Lists of Twitter handles for members of the German parliament for the 18th, 19th and 20th Bundestag were collected from an online source[§] and the Twitter Parliamentarian Database (1), resulting in a total of 820 unique Twitter handles out of 1249 members of the German Bundestag serving in the 18th, 19th or 20th Bundestag. Coverage by party and Bundestag is shown in Tab. S2.

[*] https://www.socialseer.com/resources/us-senator-twitter-accounts/
[†] https://dataverse.harvard.edu/dataset.xhtml?persistentId=doi:10.7910/DVN/MBOJNS
[‡] https://triagecancer.org/congressional-social-media
[§] https://twitter.com/i/lists/912241909002833921

party	18th Bundestag (2013-17)	19th Bundestag (2017-21)	20th Bundestag (since 2021)
total	56.1%	70.1%	81.6%
Linke	71.2%	81.7%	90.0%
Greens	85.9%	83.3%	93.2%
SPD	59.1%	73.2%	78.6%
FDP	-	87.1%	91.3%
CDU/CSU	45.3%	54.8%	70.1%
AFD	-	64.0%	84.0%

Table S2. Share of members of the German parliament that have a Twitter account and are included in the data set split by election period and party.

Lists of Twitter handles for members of the British parliament for the election periods 2015-2017, 2017-2019 and 2019 to present were collected from an online source[¶] and the Twitter Parliamentarian Database (1), resulting in a total of 726 unique Twitter handles out of 924 members of the British parliament, serving in the respective election periods. Coverage by party and election period is shown in Tab. S3.

party	2015-2017	2017-2019	since 2019
total	74.2%	87.4%	89.2%
Labour	77.6%	89.3%	93.6%
SNP	66.1%	100.0%	100.0%
LibDem	50.0%	91.7%	81.8%
Tory	74.0%	83.6%	85.5%
DUP	75.0%	80.0%	75.0%

Table S3. Share of members of the British parliament that have a Twitter account and are included in the data set split by election period and party.

The NewsGuard data base. We classified the trustworthiness of links tweeted by politicians based on the trustworthiness of the domain rather than specific items of content. We used nutrition scores provided by NewsGuard, a company that offers professional fact checking as a service and curates a large data base of domains. The trustworthiness of a domain is assessed in nine categories, each of which awards a number of points: Does not repeatedly publish false content (22), gathers and presents information responsibly (18), regularly corrects or clarifies errors (12.5), handles the difference between news and opinion responsibly (12.5), avoids deceptive headlines (10), website discloses ownership and financing (7.5), clearly labels advertising (7.5), reveals who is in charge, including any possible conflicts of interest (5), the site provides names of content creators, along with either contact or biographical information (5). In total, a domain can have up to 100 points and domains with a score of 60 or higher are labelled as "generally adhering to basic standards of credibility and transparency" (2). Similar to (3), we use this value as a threshold below which we classify a domain as "not trustworthy".

For the US, the ten most frequently linked domains that have a NewsGuard score are the Washington Post (proportion links: 3.1%, NewsGuard score: 100), New York times (3.1%, 100), Fox News (2.0%, 69.5), The Hill (1.6%, 80), CNN (1.3%, 80), Politico (1.2% 100), The Wall Street Journal (1.1%, 100), CNBC (0.9%, 95), NBC News (0.8%, 100) and NPR (0.7%, 100).

For Germany, the ten most frequently linked domains that have a NewsGuard score are Die Welt (proportion of links: 5.8%, NewsGuard score: 82), der Spiegel (5.5%, 100), die Tagesschau (3.1%, 95), die Süddeutsche (3.0%, 100), Frankfurter Allgemeine Zeitung (2.3%, 100), die Bild (2.1%, 69.5), die Zeit (2.0%, 92.5), der Tagesspiegel (1.8%, 92.5), N-tv (1.6%, 87.5) and Handelsblatt (1.5%, 100).

For the UK, the then most frequently linked domains that have a NewsGuard score are The Guardian (proportion of links: 7.2%, NewsGuard score: 100), BBC (5.2%, 95), The Times (3.0%, 100), The Telegraph (2.1%, 80), The Mirror (1.7%, 87.5), The Independent (1.7%, 100), The Daily Mail (1.2%, 64.5), NHS (0.8%, 77.5), BBC (0.7%, 95) and The Financial Times (0.7%, 100).

The average score of all sites linked by U.S. Members of Congress that have a NewsGuard score is 91.6 (12.5 SD) while the average score of linked untrustworthy sites is 40.1 (16.0 SD). The average score of all sites linked by members of the German parliament is 91.9 (12.5 SD) while the average score of linked untrustworthy sites is 24.0 (13.8 SD). The average score of all sites linked by members of the British parliament is 92.0 (10.3 SD) while the average score of linked untrustworthy sites is 39.8

[¶] https://www.politics-social.com/list/followers

(13.9 SD).

The top ten untrustworthy sites linked by U.S Members of Congress are Breitbart (38.9% of untrustworthy links), The Federalist (10.7%), Newsmax (9.7%), The Epoch Times (4.1%), Life News (2.5%), the Tennessee Star (1.6%), Red State (1.6%), One American News Network (1.6%), the Nevada Current (1.5%) and Hannity (1.5%).

The top ten untrustworthy sites linked by members of the German parliament are Journalistenwatch (15.1% or unstrustworthy links), Philosophia Perennis (11.1%), Pi News (11.1%), Achgut (10.6%), Epoch Times (10.0%), Russia Today 7.5%), Reitschuster (5.5%), Deutschland Kurier (5.1%), Freie Welt (3.7%) and Wochenblick (3.0%).

The top ten untrustworthy sites linked by members of the British parliament are Conservative Woman (23.2% of untrustworthy links), Russia Today (11.0%), English Alarabiya (7.3%), Breitbart (7.3%), Arab News (6.1%), Asian Lite (3.7%), West Monster (3.7%), Press TV (3.7%), Asian Voice (3.7%) and Gulf News (3.7%).

Investigation of domains not covered by NewsGuard. NewsGuard covers a substantial amount of domains that are linked to by politicians. After excluding links to social media platforms and search platforms (specifically we exclude twitter.com, youtube.com, facebook.com, fb.me, m.facebook.com, t.me, fb.watch, instagram.com, cards.twitter.com, google.com, google.de, yahoo.com and amp.twimg.com), the database covers 46.5% of links posted by members of the U.S. Congress, 58.8% of links posted by members of the German parliament and 39.2% of links posted by members of the British parliament. Nevertheless, the possibility remains that the NewsGuard database is biased towards including more unreliable news sources from one side of the political spectrum and that such a bias could drive our result. We therefore subjected the links that were pointing to domains *not* included in the NewsGuard database (subsequently called "missing domains") to a manual content analysis with the aim of discovering prominent news sites that are not included in the NewsGuard database. For each of the three countries, we selected all domains that individually contributed more than 0.1% to the overall volume of linked domains. This resulted in 47 domains for the US, 62 domains for Germany and 78 domains for the UK. Taken together, these domains comprise 21.2% of missing domains for the US, 31.5% of missing domains for Germany and 40.3% of missing domains for the UK.

For Germany, this analysis showed that a number of frequent domains that were not included in the NewsGuard data base were alternative links to news outlets that were included. For example, NewsGuard includes the domain "spiegel.de" but does not include the domain "spon.de", which points to the online presence of the newspaper "der Spiegel". We found 12 such instances, namely m.bild.de (alternate for bild.de), spon.de (spiegel.de), m.faz.net and zeitung.faz.net (faz.net), www1.wdr.de (wdr.de), m.tagesspiegel.de (tagesspiegel.de), app.handelsblatt.com and hbapp.handelsblatt.com (handelsblatt.com), m.focus.de (focus.de), ardmetiathek.de (tagesschau.de), amp.welt.de (welt.de) and inforadio.de (rbb.de). For the US, we found one such instance (cnn.it which is an alternate for cnn.com). After including these domains in the NewsGuard data base, 50 domains that each contributed more than 0.1% of the total link volume remained missing from the NewsGuard data base for Germany, and 46 for the US.

We then manually inspected each of the domains and assigned it a label such as "government", "personal webpage", "fundraising & petitions", "survey", "event", "blog", "cloud", "music", "social media", "podcast", "political party", "union", "university", "video", "link shortener", "video call", "news" or "dead link".

For the US, the majority of links to domains that are not included in NewsGuard point to government websites (8.3% of all links to missing domains), personal webpages (4.4%), fundraising & petition pages (3.7%) or dead links (1.1%). Among the domains that comprised more than 0.1% of the total link volume, we did not identify a single news source. While this analysis only covers 21.2% of the missing domains, we think that this analysis is a strong indicator that NewsGuard does not miss any major news sources in the US.

For Germany, the majority of links to domains that are not included in NewsGuard point to personal webpages (10.3% of all links to missing domains), political party pages (10.1%), government pages (7.4%) and news sites (1.2%). The links to news sites are distributed among four sites: queer.de (260 links), lto.de (197), topagrar.com (165) and diefreiheitsliebe.de (159). The domain queer.de is predominantly linked to by members of the Green party (194 links) while lto.de and diefreiheitsliebe.de are linked mostly by members of die LINKE. Topagrar is mostly linked to mostly by members of the FDP and the Green party. While queer.de clearly distinguishes between news and opinion, it only names responsible people for the site, not for individual articles. While clearly left-leaning, headlines don't seem to be deceptive. The Legal Tribune Online (lto.de) and topagrar.com clearly distinguish between news and opinion, names people responsible for content and does not use deceptive headlines. The site diefreiheitsliebe.de clearly indicates people responsible for content and doesn't use deceptive headlines but does not clearly distinguish between news and opinion. Given that "bild.de" is not considered untrustworthy (69.5 points), we think it is fair to conclude that diefreiheitsliebe.de, while clearly left leaning, would also not be considered "untrustworthy" by NewsGuard's standard. In summary, NewsGuard misses some significant news sources in Germany which are clearly biased in the frequency of links they receive from members of different parties, but none of the sites would be considered "untrustworthy" by NewsGuard's standards, according to our assessment.

Similar to the US, for the UK the majority of links to domains that are not included in NewsGuard point to government websites (19.3% of all links to missing domains), personal webpages (8.1%), fundraising & petitions (2.9%) or political party pages (2.4%). Different from the US, we found a small but noticeable share of links to news sites that members of the British parliament linked to but which were not included in the NewsGuard database. Specifically, we found links to the sites conservativehome.com (975), apple.news (187), yorkpress.co.uk (181) and edinburghnews.scotsman.com (178) that taken together comprised a total of 1.5% of links to missing domains. While apple.news is a news aggregator that to our knowledge does not have a political bias, conservativehome.com has a bias towards the right of the political spectrum. This is also exemplified by the fact that almost all links to the site came from members of the Conservative party (959). While it is hard to assess the site following NewsGuard's criteria, since some of the criteria (e.g. "regularly corrects or clarifies errors") require a longer observation period, an inspection of the site creates the impression that it is a politically biased but responsibly curated news site. The site clearly differentiates between comments, interviews and news, names people responsible for the content and does not seem to display deceptive headlines. We therefore conclude that it would likely not be labelled as "not trustworthy" by NewsGuard. Both yorkpress.co.uk and edinburghnews.scotsman.com are regional newspapers that are mostly linked to by members of the Labour party (167 links and 111 links, respectively). Similar to conservativehome.com, both sites clearly indicate people responsible for the content and do not use deceptive headlines. While edinburghnews.scotsman.com clearly categorizes pieces into "opinion", "news" etc., yorkpress.co.uk doesn't offer such labels. Both pages seem to focus on regional news with titles such as "Police appeal for missing North Yorkshire woman". Given our inspection of the sites, we also conclude that they would likely not be labelled as "not trustworthy" by NewsGuard. In summary, NewsGuard misses some significant news sources in the UK which are clearly biased in the frequency of links they receive from members of different parties, but they do not seem to be untrustworthy, following our assessment.

While our inspection of frequent domains not indexed in the NewsGuard data base only covers a fraction of the total number of domains that are not included in the data base, it still provides some further evidence that we are not missing any major untrustworthy news sources when using the NewsGuard data base to quantify news reliability. Based on this evidence, we cannot completely exclude the possibility of bias by sites that are not as frequently linked to, especially given the fact that the total number of links to sites that are considered "untrustworthy" is small in the UK (82) and Germany (1516). Nevertheless, the fact that we find both news sites from the left and the right side of the political spectrum that are not covered by NewsGuard provides some indication that there is no strong political bias in the inclusion criteria of the database. We recognise that the assessment criteria of NewsGuard and their interpretation might introduce a political bias into the rating of domains.

NewsGuard coverage. After excluding links to social media websites (Twitter, YouTube, Facebook, Instagram) and search engines (Google, Yahoo), the NewsGuard data base covers 46.5% of links posted by members of the U.S. Congress, 58.8% of links posted by members of the German parliament and 39.2% of links posted by members of the British parliament. The coverage over time and split by party is shown in Fig. S2.

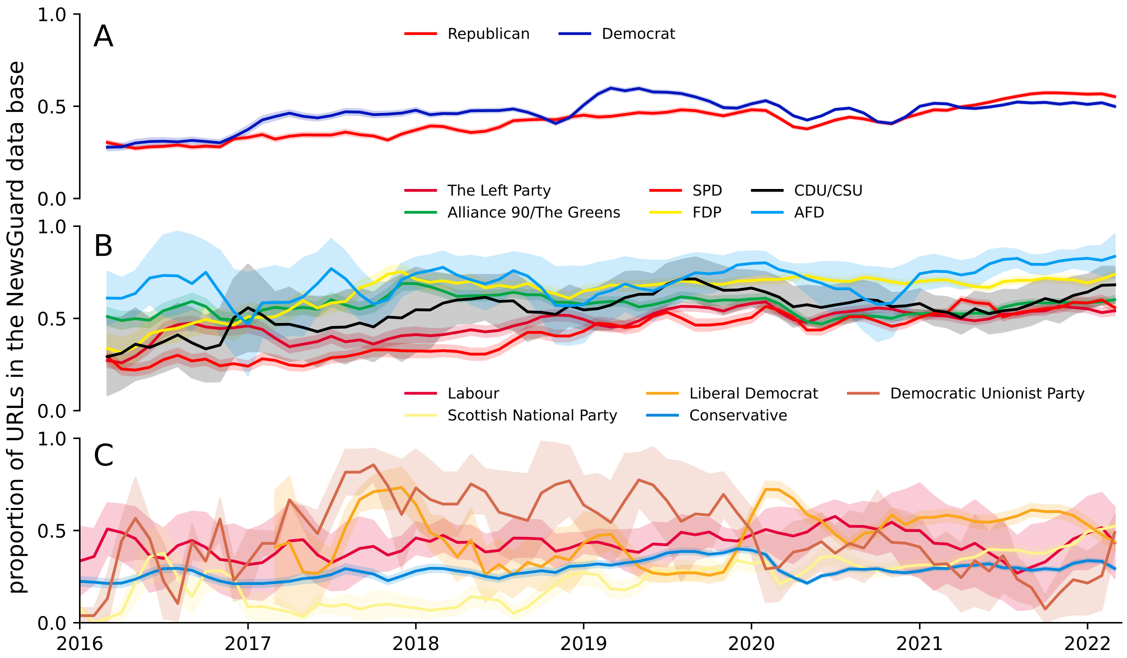

Fig. S2. Share of links covered by the NewsGuard data base for **A** links posted by U.S. Congress Members, **B** links posted by members of the German parliament and **C** links posted by members of the British parliament.

Data base of domain trustworthiness. To validate the findings using the NewsGuard data base, we compiled a list of trustworthiness ratings from a range of academic sources and fact-checking sites. Most of these sources were also used by (4). The list includes Bufale (5), Bufalopedia (6), Butac (7), Buzzfeed News (8), Columbia Journalism Review (9), Fake News Watch (10), Media Bias Fact Check (11), Politifact (12), and Melissa Zimdars (13). After merging these lists and removing duplicates, the combined list contains 4767 domains. A total of 1677 of these domains are also contained in the NewsGuard data base, as of March 1, 2022.

The main challenge in combining lists from different fact checkers lies in unifying the labels the fact checkers assign to the domains. To address this, we devise a scheme where we rate each domain on two dimension that we consider to be important to assess reliability and trustworthiness of information: "accuracy" and "transparency". We devise an accuracy scale that varies from 1 (false information) to 5 (scientific) and a transparency scale that varies from 1 (no transparency) to 3 (transparent). A more detailed description of the five accuracy levels as well as mappings of the labels of individual fact checking sites to accuracy and transparency scores is given in Tables S4 and S5. The full list of domains is available at https://doi.org/10.5281/zenodo.6536692.

After mapping all individual lists to the "accuracy" and "transparency" dimensions, we label every domain that has an accuracy score of 1 (False Information) or 2 (Clickbait) and/or a transparency score of 1 (No Transparency) as "unreliable". This results in a total of 2170 domains being labelled as "unreliable" and 2597 as "reliable". For the 1677 domains that are contained in both data bases, the Krippendorff's α between "untrustworthy" (score < 60 in NewsGuard) and "unreliable" in the independently compiled data base is 0.84, which shows a very high agreement between the two data bases.

Score	Label	Description
1	False Information	No or very little accuracy (e.g. fake news, conspiracy, satire)
2	Clickbait	Might contain smatterings of facts but is mostly misleading or clickbait
3	Biased	Mixed accuracy, half-truths, left/right bias
4	Mainstream	Low bias, mainstream media
5	Scientific	No reporting bias, scientific information

Table S4. Description of accuracy scores

Score	Label	Description
1	No Transparency	Intentionally misleading or no information about editorial process (e.g. fake news, conspiracy)
2	Mixed Transparency	Sites with (partially) transparent intention, but can still be misunderstood because of the way articles are written (e.g. bias, clickbait, satire)
3	Transparent	Sites with a transparent editorial process and legal notice (e.g. mainstream, scientific news)

Table S5. Description of transparency scores

Statistical test of differences in NewsGuard score before and after Biden's election. The NewsGuard score time series shown in Figure 1 of the main text shows a marked drop of score after president Biden's election for Republicans while the score stays stable for Democrats. To quantify this statement, we fit a linear mixed model (estimated using REML and nloptwrap optimizer) to predict NewsGuard score with party affiliation (Democrat or Republican) and time period (2016-2018 and 2020-2022). The model included time period and user as random effects. The interaction effect of time period [2020 to 2022] on party [Republican] is statistically significant and negative, suggesting a significant decrease in the trustworthiness of the information shared by members of the Republican party in the years 2020-2022 compared to 2016-2018 (beta = -4.01, 95% CI [-4.66, -3.37], t(204532) = -12.13, p $<$ .001; Std. beta = -0.32, 95 CI [-0.37, -0.27], for full results see Tab. S6).

	Dependent variable:
	NewsGuard_score
Constant	94.399***
	(0.211)
party[Republican]	−4.728***
	(0.293)
time_period[2020_to_2022]	0.191
	(0.233)
party[Republican]:time_period[2020_to_2022]	−4.015***
	(0.331)
Observations	204,540
Log Likelihood	−786,151.300
Akaike Inf. Crit.	1,572,319.000
Bayesian Inf. Crit.	1,572,400.000
Note:	*p<0.05; **p<0.01; ***p<0.001

Table S6. Linear Mixed Model coefficient table. Values in parentheses denote standard errors.

References

1. L van Vliet, P Törnberg, J Uitermark, The twitter parliamentarian database: Analyzing twitter politics across 26 countries. *PLoS one* **15**, e0237073 (2020).
2. I NewsGuard, Rating process and criteria (Internet Archive, https://web.archive.org/web/20200630151704/https://www.newsguardtech.com/ratings/rating-process-criteria/) (2020) Accessed: 2022-04-20.
3. S Bhadani, et al., Political audience diversity and news reliability in algorithmic ranking. *Nat. Hum. Behav.* pp. 1–11 (2022).
4. R Gallotti, F Valle, N Castaldo, P Sacco, M De Domenico, Assessing the risks of 'infodemics' in response to covid-19 epidemics. *Nat. Hum. Behav.* **4**, 1285–1293 (2020).
5. Bufale, The black list (https://www.bufale.net/) (2022) Accessed: 2022-05-01.
6. Bufalopedia, Un catalogo di indagini e risorse antibufala (https://bufalopedia.blogspot.com/p/siti-creatori-di-bufale.html) (2020) Accessed: 2022-05-01.
7. Butac, The black list (https://www.butac.it/the-black-list/) (2022) Accessed: 2022-05-01.
8. B News, Inside the partisan fight for your news feed (https://www.buzzfeednews.com/article/craigsilverman/ inside-the-partisan-fight-for-your-news-feed) (2017) Accessed: 2022-05-01.
9. CJ Review, CJR index of fake-news, clickbait, and hate (http://web.archive.org/web/20210720140548/https://www.cjr.org/fake-beta) (2021) Accessed: 2022-05-01.
10. FN Watch, Fake news watch (https://web.archive.org/web/20180213181029/http://www.fakenewswatch.com/) (2018) Accessed: 2022-05-01.
11. MBF Check, Media bias fact check (https://mediabiasfactcheck.com/) (2022) Accessed: 2022-05-01.
12. Politifact, Politifact's guide to fake news websites and what they peddle (https://www.politifact.com/article/2017/apr/20/politifacts-guide-fake-news-websites-and-what-they/) (2017) Accessed: 2022-05-01.
13. M Zimdars, My "fake news list" went viral. but made-up stories are only part of the problem. (https://www.washingtonpost.com/posteverything/wp/2016/11/18/my-fake-news-list-went-viral-but-made-up-stories-are-only-part-of-the-problem/) (2016) Accessed: 2022-05-01.
